# Women’s experiences of prison-based mental healthcare: a systematic review of qualitative literature

**DOI:** 10.1108/IJPH-09-2021-0091

**Published:** 2022-02-24

**Authors:** Ann-Marie Bright, Agnes Higgins, Annmarie Grealish

**Affiliations:** Department of Nursing and Midwifery, University of Limerick, Limerick, Ireland; School of Nursing and Midwifery, Trinity College Dublin, Dublin, Ireland; Department of Nursing and Midwifery, University of Limerick, Limerick, Ireland and Florence Nightingale Faculty of Nursing, Midwifery and Palliative Care, King’s College London, London, UK

**Keywords:** Mental health, Women prisoners, Prison, Service provision, Qualitative synthesis, Women’s experiences

## Abstract

**Purpose:**

The rate of female committals to prison has grown rapidly in recent years. Women in prison are likely to have trauma histories and difficulties with their mental health. This paper aims to synthesise the findings of qualitative literature to gain a deeper understanding of the experiences of women in the context of prison-based mental health care.

**Design/methodology/approach:**

A systematic search of five academic databases, Cumulative Index to Nursing and Allied Health Literature, Applied Social Sciences Index and Abstracts, Psychological Information Database (PsycINFO), Excerpta Medica DataBASE (EMBASE) and Medline, was completed in December 2020. This study’s search strategy identified 4,615 citations, and seven studies were included for review. Thomas and Harden’s (2008) framework for thematic synthesis was used to analyse data. Quality appraisal was conducted using the Joanna Briggs Institute Checklist for Qualitative Research (Lockwood *et al.*, 2015).

**Findings:**

Four analytic themes were identified that detail women’s experiences of prison-based mental health care: the type of services accessed and challenges encountered; a reduction in capacity to self-manage mental well-being; the erosion of privacy and dignity; and strained relationships with prison staff. There is a paucity of research conducted with women in the context of prison-based mental health care. The findings suggest there is a need for greater mental health support, including the need to enhance relationships between women and prison staff to promote positive mental health.

**Originality/value:**

To the best of the authors’ knowledge, this is the first systematic review conducted on the experiences of women in the context of prison-based mental health care.

## Introduction

Since 2000, the number of women in prison has increased by approximately 53%, resulting in a worldwide female prison population estimated at 714,000 (Walmsley, 2017). Internationally, prison populations and committal rates vary. The USA has seen a 700% increase in female incarceration between 1980 and 2019, with approximately 222,455 women in prison and jail between the years 2018 and 2019 (Carson, 2020; Zeng, 2020). However, Australia saw a 10% reduction of women in prison from 2019 to 2020, which may be the result of pandemic restrictions implemented in various states and territories (Australian Bureau of Statistics, 2020). In the UK, women account for 4.1% of the overall prison population (World Prison Brief, 2021), where the average female prison sentence is 11.3 months (Ministry of Justice, 2019), and in Ireland, the average duration of prison sentences are three months or less (Irish Prison Service, 2019a). In the UK, it is estimated that 73% of women serving sentences of 12 months or less are reconvicted within a year of release (Prison Reform Trust, 2021). The effectiveness of short prison sentences has been debated as they are less effective at reducing reoffending when compared with suspended or community sentences (O’Donnell, 2020; Gascón, 2020). Indeed, the prison has a negative impact on women in the context of mothering, homelessness and employment (O’Malley and Devaney, 2016; Powell *et al.*, 2017; Prison Reform Trust, 2021) and with only two women’s prisons in Ireland and 12 in the UK, many women are detained far from home, impacting on the visitation of children and families (Irish Prison Service, 2021; Ministry of Justice, 2021a).

Mental health problems are overrepresented in the female prison population; approximately 80% have a mental health diagnosis (World Health Organisation, 2021). Women in prison are five times more likely to experience mental health difficulties than women in the general population (Tyler *et al.*, 2019). Prevalence rates for psychotic illness among women in prison are estimated at 3.9%, major depression at 14.1%, alcohol misuse at 10%–24%, drug misuse at 30%–60% (Fazel *et al.*, 2016) and post-traumatic stress disorder at 21.1% (Baranyi *et al.*, 2018). Women in prison are susceptible to self-harm, with approximately 30% engaging in self-harming behaviours (Ministry of Justice, 2017; Irish Prison Service, 2020; Ministry of Justice, 2021b). In addition, women in prison are up to 20-times more likely to die by suicide and 36 times more likely to die by suicide one-year post-release when compared with those in the general population (Fazel and Benning, 2009; Pratt *et al.*, 2010). Women often experience domestic, physical, emotional and sexual abuse prior to imprisonment giving rise to complex and often unresolved trauma that may be criminogenic (Gunter, 2012; Alves *et al.*, 2016). A correlation exists between trauma and the development of mental health difficulties (Karlsson and Zielinski, 2018; Jewkes *et al.*, 2019), which are factors for a range of adverse outcomes in prison and on release, including being victims of violence and assault (Caravaca-Sánchez *et al.*, 2014) and reoffending (Baillargeon *et al.*, 2009).

Recently, there has been an increased call for gender-specific mental health care in prisons (Public Health England, 2018). Internationally, gender-specific care is advocated for in the Bangkok Rules (United Nations Office on Drugs and Crime, 2012), whereas the Kyiv Declaration made recommendations to review policies and services to meet the needs of women in prison (United Nations Office on Drugs and Crime, 2009). Mental health services have adopted trauma-informed approaches (Muskett, 2013; Department of Health, 2020), which should also inform prison-based mental health care.

A recent meta-synthesis by Ratcliffe and Stenfert Kroese (2021) provides an overview of women’s experiences of forensic settings in the UK but does not include prison settings. Other systematic reviews of mental health interventions for prisoners tend to focus on medication and psychotherapy (Fazel *et al.*, 2016), CBT and mindfulness-based therapy (Yoon *et al.*, 2017) and interventions for early adults (Givens *et al.*, 2021), but neglect to consider gender-specific differences or make gender-specific recommendations. Given that the rate of women being sentenced to prison is growing more rapidly than for men (Central Statistics Office, 2019), there is a greater need to focus on the female prison population. Therefore, this review aims to synthesise findings from qualitative research conducted with women in prison to identify the state of the evidence in this area and gain a deeper understanding of prison-based mental health care from women’s perspectives.

## Methods

This systematic review is reported in accordance with the preferred reporting items for systematic reviews and meta-analyses statements (PRISMA) (Page *et al.*, 2021). The study protocol is registered with the international prospective register of systematic reviews (PROSPERO) (CRD42021240407). A population, concept and context (PCC) framework was used to guide the selection of terms used in the search strategy and to formulate the following research question:

RQ1. What are the experiences of women in prison in the context of prison-based mental health care?

### Search strategy

A preliminary search of the Cochrane Library, Google Scholar, Open Grey database and PROSPERO was undertaken to ensure no other related systematic reviews had been conducted. A systematic search of five databases was conducted in December 2020; Cumulative Index to Nursing and Allied Health Literature, Medline, Psychological Iinformation Database, Excerpta Mmedica DataBASE and Applied Social Sciences Index and Abstracts. There was no restriction on language, geographical origin or publication date owing to a dearth of research in this subject area. The terms in the PCC framework were searched as keywords, topics, Medical Subject Headings (MeSH) and combined using Boolean operators (Table 1). The search protocol was developed with a librarian and tested against preselected articles. Forward citation tracking of retrieved articles was conducted by hand searching reference lists of returned articles to maximise the yield of relevant papers.

### Eligibility criteria

Studies were included if the primary research approach was qualitative; they reported on women in prison who had accessed prison-based health care for mental ill-health and sampled women were over 18 years with self-reported or diagnosed mental health problems. Studies were excluded if they did not report primary qualitative narratives; it was not possible to disaggregate data of female participants in studies that included male participants, and they used quantitative designs, expert opinion or consensus statements.

### Study selection

All citations were uploaded to Zotero for de-duplication and exported to Colandr (Cheng *et al.*, 2018) for title/abstract and full-text screening. Two authors (AB and AG) independently assessed titles and abstracts against the eligibility criteria, and articles were excluded by agreement with all authors. Full-text copies of all remaining articles were independently screened by (AB, AH and AG) against eligibility criteria. Any conflict regarding the eligibility criteria of individual studies was resolved by discussion.

#### Quality appraisal.

Quality appraisal of the included studies was independently undertaken by AB and AH using the Joanna Briggs Institute Checklist for Qualitative Research (Lockwood *et al.*, 2015) to assess the methodological quality, rigour, trustworthiness, relevance and results. This tool does not recommend the use of a scoring system; instead, it contains 10 questions rated as “yes”, “no”, “unclear” or “not applicable”. The quality weightings of the included studies (Table 2) were considered when reporting and discussing the results.

#### Data extraction and analysis.

A Microsoft Excel data extraction form was developed and piloted for extracting information from each study. This included author, year, country of origin, study aim, sample achieved, population characteristics and categories related to recruitment and data collection. Information related to how women accessed mental health care in prison, women’s experiences of prison-based mental health care, barriers and enablers to accessing prison-based mental health care and reported themes and recommendations were extracted. Thematic synthesis using the Thomas and Harden (2008) framework was undertaken on qualitative data. Studies were read and re-read by all team members and qualitative data relevant to the question was extracted and coded inductively, keeping the synthesis close to the original findings. Finally, descriptive themes were analysed and, through an iterative process of review and inference, analytic themes were identified with reference to the review question.

## Results

### Search outcomes

**Figure 1:**
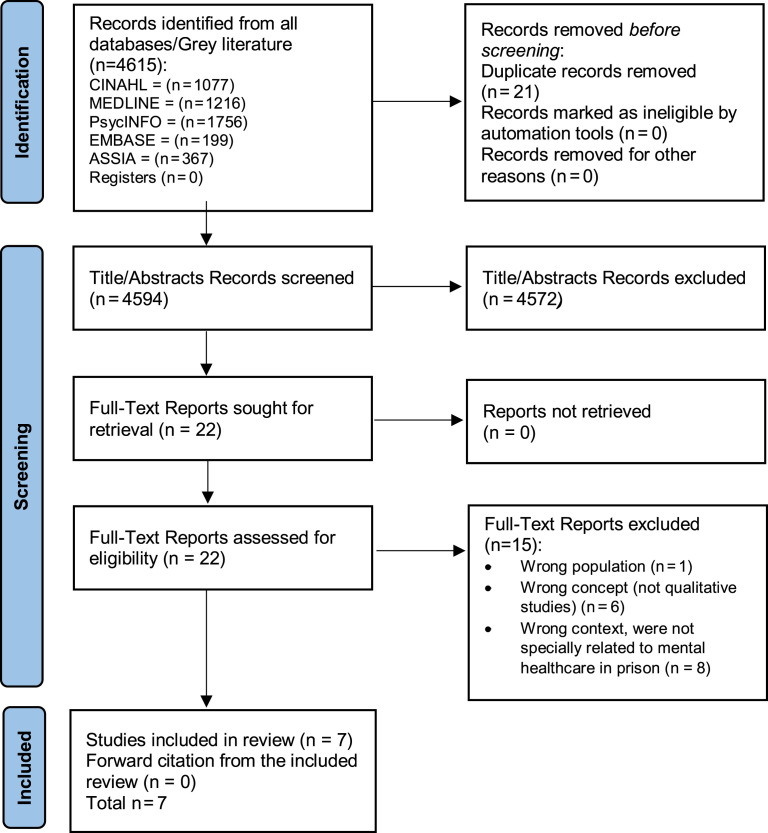
PRISMA flowchart

The number of studies that identified study selection against the eligibility criteria and reasons for exclusion is depicted in Figure 1. The search strategy yielded 4,615 studies from five databases and none from grey literature sources. After removing duplicates (*n* = 21), 4,594 studies were retained for the title and abstract screening. A total of 22 citations met the eligibility criteria for full-text screening, from which a final total of seven papers were eligible for this review.

### Study characteristics

Table 3 presents a summary of the characteristics of the seven included studies that were conducted between 2008 and 2018. One study focused specifically on psychiatric services (Jacobs and Giordano, 2018), with the remaining studies focusing on general health care or different aspects of mental health such as self-harm, suicide and medication. Two studies (Bowen *et al.*, 2009; Jacobs and Giordano, 2018) were conducted on female and male participants; however, only data related to female participants were extracted. Three studies were conducted in England (Plugge *et al.*, 2008; Kenning *et al.*, 2010; Caulfield, 2016), two across England and Wales (Bowen *et al.*, 2009; Marzano *et al.*, 2011) one in Canada (Ahmed *et al.*, 2016) and one in the USA (Jacobs and Giordano, 2018). Study designs were mostly qualitative; Jacobs and Giordano (2018) used an inductive qualitative approach by using techniques from Interpretive Phenomenology and Constructivist Grounded Theory; Bowen *et al.* (2009) used a narrative approach. Four studies (Plugge *et al.*, 2008; Kenning *et al.*, 2010; Caulfield, 2016; Ahmed *et al.*, 2016) were qualitative descriptive. Marzano *et al.* (2011) used mixed methods from which only qualitative data were extracted.

Six studies (Plugge *et al.*, 2008; Bowen *et al.*, 2009; Kenning *et al.*, 2010; Marzano *et al.*, 2011; Caulfield, 2016; Jacobs and Giordano, 2018) used semi-structured, one-to-one interviews to collect data. Ahmed *et al.* (2016) used focus groups. In addition to interviews, Plugge *et al.* (2008) used focus groups (*n* = 6) and Bowen *et al.* (2009) added non-participant observation to complement their data collection. Marzano *et al.* (2011) and Plugge *et al.* (2008) conducted interviews in a private room in a prison setting with no staff present. Caulfield (2016) does not report the interview setting, only that the participant and researcher were present during the interview. Kenning *et al.* (2010), Jacobs and Giordano (2018) and Bowen *et al.* (2009) did not report where their interviews were held.

#### Participant characteristics.

The collective review sample was 255 female participants, and individual female study sample sizes ranged from 4 to 120. All participants were currently serving a prison sentence apart from those in Jacobs and Giordano (2018), who were women under court supervision after recent detention. Participants in Ahmed *et al.* (2016) were all on remand; Marzano *et al.* (2011) reported that in their total sample, 25 participants (*n* = 25) were on remand whilst 95 participants (*n* = 95) were sentenced. Plugge *et al.* (2008) reported six participants (*n* = 6) in the focus groups were on remand, with 31 participants (*n* = 31) sentenced; however, no status was provided for interview participants. Kenning *et al.* (2010) reported 11 participants (*n* = 11) were sentenced whilst four (*n* = 4) were on remand. Bowen *et al.* (2009) and Caulfield (2016) did not report on participant status.

Participant ages ranged from 18 to 60 years, and of the studies that reported a mean age, the means ranged from 25.5 to 45 years. Six of the included studies reported some detail on participant ethnicity. However, as not all studies reported in the same manner, it is not possible to provide an aggregate. For the six studies that did report ethnicity (Plugge *et al.*, 2008; Kenning *et al.*, 2010; Marzano *et al.*, 2011; Caulfield, 2016; Ahmed *et al.*, 2016; Jacobs and Giordano, 2018), participants were described as White, British Black, Black Caribbean, Aboriginal, African, African/Caribbean and mixed-race. Two studies reported specifically on the mental health problems experienced by women; Caulfield (2016) reported participants had mental health and emotional difficulties prior to their current sentence. Participants (*n* = 15) in Kenning *et al.* (2010) all engaged in self-harming behaviours of which 11 (*n* = 11) had a history of self-harm outside of prison. Although Bowen *et al.* (2009) and Jacobs and Giordano (2018) reported on mental health problems, they did not provide a breakdown between male and female participants.

### Quality of studies

Overall, the body of evidence was of mixed quality, and the results of the methodological quality assessment are outlined in Table 4. In all but one study, Jacobs and Giordano (2018) reported receiving ethical approval. Jacobs and Giordano (2018) specified the study was compliant with ethical standards of the institutional and national research committees and the 1964 Helsinki declaration. Although the studies provided variable descriptions on the data collection methods, none made explicit the philosophical perspective underpinning the research, and the exact methodology was not described by four of the included studies (Plugge *et al.*, 2008; Kenning *et al.*, 2010; Caulfield, 2016; Ahmed *et al.*, 2016). This led to the reviewers having to make inferences about the methodology from the descriptions provided. None of the studies provided a clear statement that located the researcher culturally or theoretically. Only Jacobs and Giordano (2018) addressed the influence of the researcher on the research and vice-versa. The sample size of females in some studies was small; additionally, as not all studies reported on participant demographics, it was difficult to know if an adequate representation of women in prison was achieved.

### Four analytic themes

**Figure 2:**
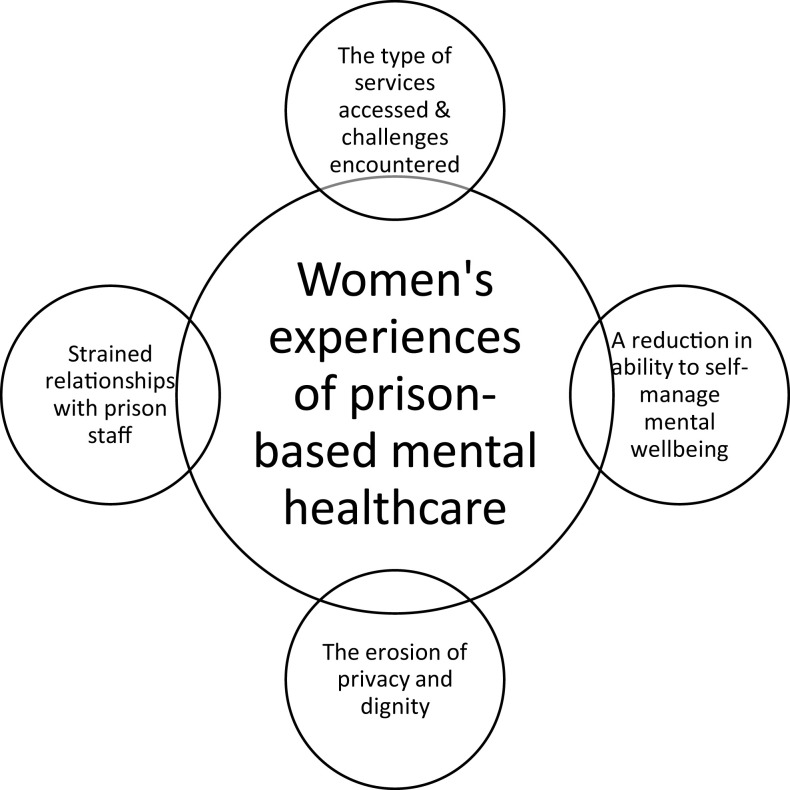
Thematic summary diagram

Four main themes were elected from our findings:

1. the type of services accessed and challenges encountered;
2. a reduction in ability to self-manage mental well-being;
3. the erosion of privacy and dignity and;
4. strained relationships with prison staff. Figure 2 presents an overview of women’s experiences of prison-based mental health care.

Table 4 shows the grouped findings from the studies, and quotations are included to illustrate the theme from study participants.

#### Theme 1 – the type of services accessed and challenges encountered.

The first analytic theme describes women’s experiences of accessing mental health services whilst in prison.

None of the studies made explicit the mental health services available to women, although the women themselves mentioned in their narratives Social Workers (Bowen *et al.*, 2009; Jacobs and Giordano, 2018), Psychologists (Bowen *et al.*, 2009; Caulfield, 2016; Jacobs and Giordano, 2018), Psychiatrists (Caulfield, 2016), Clinical Staff (Marzano *et al.*, 2011; Jacobs and Giordano, 2018), Nurses (Plugge *et al.*, 2008; Bowen *et al.*, 2009), Doctors (Plugge *et al.*, 2008; Bowen *et al.*, 2009; Caulfield, 2016), Occupational Therapists (Bowen *et al.*, 2009) and Counsellors (Caulfield, 2016). The importance of timely and regular access to mental health services in prison was highlighted by women in some studies. Women described positive health outcomes from being able to attend counselling while in prison and being able to access medication on a regular basis (Caulfield, 2016). Similarly, women described psychiatric services as helpful with their mental health problems because they “gave me medications” (Jacobs and Giordano, 2018).

Conversely, women in Ahmed *et al.’s* (2016) study described several challenges in accessing mental health care; women reported not knowing what services were available, which the authors suggest was the result of poor health literacy, and some women were unable to read health information material and felt embarrassed or fearful when asking for help. Another challenge was the application process used to access health care, which required women to complete a form stating why they needed to see particular health-care professional. This process was described as taking “too long”, with significant delays (Plugge *et al.*, 2008). Some applications were reported to be refused or not responded to, resulting in a decline in women’s health status (Ahmed *et al.*, 2016). Being refused access to health practitioners or witnessing care not being provided was described as “discouraging and tiresome”; thus, women felt that asking for help was futile, and they chose not to write subsequent requests (Ahmed *et al.*, 2016). Women transferred within the prison system described a lack of continuity of care, as prescriptions were sometimes changed when women moved prisons, and in the context of drug detoxification, women describe becoming “very unwell” as a result of these practices (Bowen *et al.*, 2009).

#### Theme 2 – a reduction in ability to self-manage mental well-being.

The second analytic theme outlines how women experienced a reduction in their ability to self-manage their mental well-being because their civil liberties had been curtailed.

A prison sentence results in the removal of an individual’s freedom, thus reducing capacity to self-manage. Women describe not being able to take a taxi to a medi-centre, which impacted their ability to manage their own health care needs (Ahmed *et al.*, 2016). Women reported that their usual self-medication practices were curtailed, resulting in disruptions to the strategies they used to control symptoms (Bowen *et al.*, 2009). Women also reported “reluctance” to engage with medical staff if dose discrepancies were noted out of fear that medication would be completely discontinued (Bowen *et al.*, 2009). Changes were made to their prescriptions by prison doctors without providing a rationale or without consultation, a practice that increased women’s anxiety about how they would cope (Bowen *et al.*, 2009). Similarly, women reported their community prescriptions were sometimes discontinued despite informing prison health-care staff of existing dose regimens, and in the context of drug detoxification, this resulted in women being unable to sleep or manage symptoms of withdrawal (Plugge *et al.*, 2008). Some participants reported being “bullied” by other prisoners for the medication (Marzano *et al.*, 2011). When a participant asked for counselling in addition to the medication they were refused as “the doctor said that anti-depressants would help” further compounding this lack of self-management (Caulfield, 2016).

The purpose of self-harming behaviours used by women in prison was described as a means of coping with emotions and as a way of relieving frustration and pain, indicating a reduction in adaptive self-management skills (Kenning *et al.*, 2010). Precipitating factors related to self-harming behaviours consisted of being denied certain requests, being put on “basic regime” and feelings of “disempowerment from being in prison” (Kenning *et al.*, 2010). These findings demonstrate the extremes women engaged with to feel a sense of mastery over their lives and experiences.

#### Theme 3 – the erosion of privacy and dignity.

The third analytic theme describes the erosion of women’s privacy and dignity and how prison staff, as well as the women themselves, were the perpetrators of a reduction of privacy and dignity.

Women’s privacy was eroded by nursing staff when medical problems were discussed in prison corridors and at medication “hatches” (Plugge *et al.*, 2008). In addition, nursing staff eroded privacy by maintaining a presence during medical consultations and by acting as “gatekeepers” and “bodyguards” when women met with doctors. Women’s dignity was eroded when nurses intervened to “interpret [your] symptoms for you” as opposed to letting women recount their own stories (Plugge *et al.*, 2008). However, the erosion of privacy was not unique to health-care staff, with women themselves being complicit by reading health care application forms belonging to their peers (Ahmed *et al.*, 2016). Women describe reading health care applications that were left “on the panel” and question why a woman would detail sensitive information on these forms with the knowledge that anyone could read them (Ahmed *et al.*, 2016). Women described being “looked down” on by prison staff because they were in prison and that care provided was sub-standard compared to community care (Plugge *et al.*, 2008). In the context of dignity, health information was described as being *too* simple and almost patronising, by not communicating the serious nature of the illnesses within the information provided and by not taking into account the abilities of the women to comprehend this information (Ahmed *et al.*, 2016).

#### Theme 4 – strained relationships with prison staff.

This final analytical theme describes women’s views about their relationships with prison staff and the impact of these relationships on their perceptions of prison-based mental health care.

Descriptions of interactions reflected poor relationships between the women and those providing care. Relationships were described as “anti-therapeutic” in the context of prescribing medication (Bowen *et al.*, 2009). Women reported that requests to have medications adjusted were generally ignored, resulting in strained relationships. Similarly, women reported that clinicians “did not care” about their issues, and when endeavouring to access the health care, they felt they were “sloughed off” and were “dissatisfied” with responses received from prison staff (Ahmed *et al.*, 2016). Women perceived that relationship difficulties were a precipitating factor to self-harming behaviours and that “being treated better by prison staff” may have prevented these acts (Marzano *et al.*, 2011). Staff were described as “cold” and “desensitised” (Kenning *et al.*, 2010), and although women acknowledged that prison staff work in difficult environments, they also described prison health-care staff as “unqualified”, “unprofessional” and “rejects”, reflecting the degree to which relationships were strained (Plugge *et al.*, 2008).

## Discussion

To our knowledge, this is the first systematic review to synthesise qualitative literature that reports the experiences of women in the context of prison-based mental health care. The findings of this review illustrate the lack of research conducted in this area. Although the seven included studies crossed a 10-year time span and did not elicit in-depth narratives from the women, the findings do provide some insights.

Women in prison are more likely to participate in mental health treatment programmes than their male counterparts (Crittenden and Koons-Witt, 2017). However, findings from this review suggest they are less likely to be offered or have access to treatment programmes. This review highlights the number of challenges women experienced accessing quality services and mental health interventions in a timely and co-ordinated manner, which parallels those seen by women with a history of incarceration in a community context (Mongelli *et al.*, 2020). Bartlett and Hollins (2018) assert women’s criminal behaviours should be seen in the context of trauma and vulnerabilities and that prison should be a truly rehabilitative process where quality mental health care can be provided. This review reveals that women are disempowered from managing their mental health, which may impact their ability to manage their mental and physical health when released from prison. This raises an important question: how can this be considered rehabilitative? Recovery is a multi-definitional concept which, at its core, speaks to individuals developing new meaning and purpose in their lives (Anthony, 1993) and provides a framework for service delivery. Leamy *et al.* (2011) identified five recovery themes essential for recovery to occur; these include connectedness, hope, identity, meaningful role and empowerment. Therefore, it is essential that women are empowered to manage their own mental health whilst in prison so that they can contribute to their own personal recovery and rehabilitation. This can be supported by strengths and skills-building activities and also by increasing self-esteem and self-efficacy (Leamy *et al.*, 2011).

As a direct result of being in prison, an individual loses some of their fundamental human rights, particularly the right to liberty (United Nations, 1948). However, not all human rights are lost, and this is particularly relevant in respect of the right to privacy. Confidentiality is one of the core ethical principles underpinning health care (Beauchamp and Childress, 2013), irrespective of the context of service provision. Although the findings demonstrate how prison can provide an opportunity to access specialist mental health services, which can have a positive impact on mental well-being (Durcan and Zwemstra, 2014), the review also highlights that women’s applications for health care access were read by others and private information was discussed in corridors and at hatches. This eroded women’s right to privacy and resulted in a reluctance to seek help. Having access to quality mental health services is essential for maintaining mental well-being and is considered a fundamental human right by the World Health Organisation (2017).

Considering prisoners are a vulnerable population, it is of great concern that interpersonal difficulties with prison staff were cited by participants in five of the seven included studies (Plugge *et al.*, 2008; Bowen *et al.*, 2009; Kenning *et al.*, 2010; Marzano *et al.*, 2011; Ahmed *et al.*, 2016). Women described staff as unresponsive to their needs, which led to a deterioration in women’s mental health and the creation of a cycle of silence. When access to mental health care was refused or interventions not provided, women’s self-efficacy was diminished; subsequently, they chose not to seek further help or question discrepancies with medication dosages (Plugge *et al.*, 2008; Bowen *et al.*, 2009). Consequently, this further exacerbated their mental health problems and led to strained relationships with prison staff. There appear to be differences between women’s experiences of prison staff, as highlighted by this review’s findings, compared with women’s experiences of forensic-setting staff. Women reported they felt validated, humanised and understood in a forensic setting (Ratcliffe and Stenfert Kroese, 2021), which may suggest that the “for treatment” focus of a forensic setting is more acceptable to women than the “as punishment” focus of the prison. There is no doubt that prison staff work in busy environments, and parallels can be drawn between staff working in acute mental health services where burnout and compassion fatigue can occur as a result of under-resourced working environments, excessive workload and a lack of opportunity (Short *et al.*, 2009; Johnson *et al.*, 2018). However, irrespective of these challenges, prison staff need to consider confidentiality as a norm rather than an exception.

Those working in a prison environment are ideally placed to advocate for prompt access to mental health services and the development of policies and systems that ensure quality mental health service provision (Irish Prison Service, 2019b; Scottish Prison Service, 2021). Mental health education for prison staff may help to enhance their understanding of how mental health problems manifest and specific vulnerabilities. It may also enable a more compassionate and respectful approach to service delivery (Irish Prison Service, 2020). Education may also enhance relationships between women in prison and prison staff, with better mental health outcomes for the women and improved working environments for the staff.

Poverty and inequality are common among prisoners and contribute to mental ill-health and, whilst a link between mental health and criminality has been contested (Halle *et al.*, 2020), this combination results in the marginalisation of those impacted (Webster and Qasim, 2018). Marginalised women experience poorer mental health outcomes (Department of Justice and Equality, 2017) and, as the majority of the studies located did not elicit in-depth data on women’s experiences of prison-based mental health care, this also suggests that women in prison are not only marginalised by society, but also by the research community. Roberts (2000) posits that narratives add value to what is known and understood about the lived experience. Hence, there is a clear need for further qualitative research in this context, both to inform policy and service provision and to let the voices of women in prison be heard.

## Limitations

This review represented the experiences of 255 women in prison from various backgrounds and geographic locations, yet it found similarities in their experiences in prison and their concerns related to prison-based mental health care. However, the review needs to be read in the context of the following limitations. There was a significant lack of qualitative studies conducted on this topic that has impacted not only the findings of the review, but also the recommendations for policy and practice. Given the lack of diversity represented in the combined population of participants and the small study sample sizes, the transferability of the findings to all women in prison is not possible. Another issue that needs to be acknowledged is that all the studies were conducted in high-income countries, and consequently, the narrative experiences may not be reflective of the prison experiences of women in low-income countries or indeed of the various cultural contexts associated with prison systems internationally. As no date limiters were set, dated research has been included. Subsequently, the review team was unable to provide commentary on the contemporary position of women in prison, as their experiences may have been shaped by any service reform or policy change that has occurred in the intervening years. Although no geographical or language limiters were set, the fact that only studies published in the English language and from high-income countries were located has potentially introduced a bias. As the review was designed to include all relevant research, the quality appraisal undertaken was not to exclude studies. Thus, studies that had lower ratings on quality were included. Finally, the findings may have been shaped by the review team’s experiences as practitioners and researchers in mental health, and therefore, what was deemed relevant may be a result of professional training, standards and perspective.

Future research would benefit from further qualitative studies on diverse populations that focus exclusively on women’s experiences of prison-based mental health care. It would be beneficial for future research to consider participant status as women’s experiences may be shaped according to them being sentenced or on remand. Future research should also provide more contextual information on the nature of the mental health services available to women.

## Conclusion

This systematic review has highlighted an urgent need for the international implementation of gender-specific mental health care in prisons as well as a need for further studies that explore the experiences of women using mental health services in prison. Despite having been found guilty of a crime punishable by a custodial sentence, women often have histories of trauma and vulnerabilities. Therefore, prison should be an experience where those serving time can, if they choose to, develop new ways of navigating their social worlds outside of the sphere of crime and where they can access quality mental health interventions if required. If prison is to be a truly rehabilitative process, women need to encounter staff that embodies the humanistic principles of empathy and unconditional positive regard.

## Figures and Tables

**Table 1:** PCC Framework

**Population** Women prisoners with mental ill-health	Woman OR women OR female OR prisoner* OR imprison* OR convict* OR inmate* OR offender* OR sentence* OR jail OR correctional OR criminal justice OR incarcerat* OR criminal* OR remand OR felon OR penitentiar* OR detain* OR penal OR mental health OR mental illness OR mental disorder OR mental health problem* OR mental ill-health OR mental ill health OR psychiatric problem* OR psychiatric disorder* OR psychiatric illness* OR psychological problem* OR psychological issue* OR psychological illness* OR symptom* AND
**Concept** Qualitative experiences	Experience* OR attitude* OR perceptions OR opinions OR thoughts OR beliefs OR satisfaction OR satisf* OR qualitative OR qualitative research AND
**Context** Mental health care in prison	Mental health service OR mental health care OR intervention* OR care OR support* OR help OR treatment

**Table 2:** Quality appraisal

Joanna Briggs Institute Check List for Qualitative Research: Questions	Plugge *et al.* (2008)	Bowen *et al.* (2009)	Kenning *et al.* (2010)	Marzano *et al.* (2011)	Caulfield (2016)	Ahmed *et al.* (2016)	Jacobs and Giordano (2018)
Is there congruity between the stated philosophical perspective and the research methodology?	U	U	U	U	U	U	U
Is there congruity between the research methodology and the research question or objectives?	U	Y	U	Y	U	U	U
Is there congruity between the research methodology and the methods used to collect data?	Y	Y	Y	Y	Y	Y	Y
Is there congruity between the research methodology and the representation and analysis of data?	Y	Y	Y	Y	Y	Y	Y
Is there congruity between the research methodology and the interpretation of results?	Y	Y	Y	Y	Y	Y	U
Is there a statement locating the researcher culturally or theoretically?	N	N	N	N	N	N	U
Is the influence of the researcher on the research, and vice-versa, addressed?	U	U	U	N	N	U	Y
Are the participants, and their voices adequately represented?	Y	Y	N	Y	Y	U	N
Is the research ethical according to current criteria or, for recent studies, and is there evidence of ethical approval by an appropriate body?	Y	Y	Y	Y	Y	Y	N
Do the conclusions drawn in the research report flow from the analysis, or interpretation, of the data?	Y	Y	Y	Y	Y	Y	Y

**Table 3:** Study characteristics

Author, year and Country	Ethical approval	Study aim and methodology	Data collection methods	Recruitment methods	Data collection process	Data analysis	Findings/key themes
Plugge *et al.* (2008) Southern England	South East Multicentre Research Ethics Committee (04/MRE01/14)	To explore women prisoners’ experiences of health care provision as part of a wider project examining the health impact of imprisonment on women. Qualitative descriptive (inferred)	Focus groups ×6: Participants (*n* = 37) Audio recorded Semi-structured, individual interviews: Participants (*n* = 12) Audio recorded	Focus groups –purposive sampling using local inmate directory Interviews – convenience sampling from women participating in a longitudinal questionnaire over a 3-month period	Location: focus groups – held in a private room in the prison health centre Interviews – held in private room in the prison health centre	Thematic analysis (framework not reported) N-vivo (v7)	Application process to access health care is a protracted process. Nurses perceived as “gatekeepers” and “bodyguards” Health-care staff competence questioned by participants “rejects”, “unprofessional”, unqualified” Lack of privacy when discussing health care issues
Bowen *et al.* (2009) England & Wales	South East Multi-Centre Research Ethics Committee	Drawing on narrative accounts of prisoners and the staff they must negotiate, this paper considers the prescribing and taking of medication related to the management of mental health problems in a prison context. Qualitative – narrative synthesis	Semi-structured, individual interviews: Participants (*n* = 39) Audio recorded Non-participative observation	Purposive sample of: Those known to have a recent history of mental disorder Were currently withdrawing from drug/alcohol misuse Had experience of processes for the management of suicide/self-harm Had been in prison for at least two weeks and less than approximately 8 months	Interview location: not stated Interview privacy: Not stated Non-participative observation location: Reception, induction, residential, in-patient and detoxification units. Participant observation duration: between 2 and 7 h in each location.	Thematic analysis closely following Miles and Huberman (1984) “Drawing Valid Meaning from Qualitative Data: Toward a Shared Craft”	Autonomy – lack of autonomy impacted the women’s ability to self-manage Lack of personal control over medication taking Changes to medication regimes disruptive to routines and cause anxieties, confusion and distress Anti-therapeutic relationships between participants and staff
Kenning *et al.* (2010) North West England Ealing and West London Mental Health Trust Ethics Committee	Central and Eastern Cheshire Primary Care Trust.	To compare attitudes of women prisoners who self-harm with the attitudes and perspectives of different prison staff and to examine the possible impact on the delivery and development of prison services. Qualitative descriptive (inferred)	Semi-structured, individual interviews Participants (*n* = 15) Audio recorded	Purposive sample according to where the women prisoners were housed within the prison.	Interview location: not stated	Grounded Theory	Health care staff were positively perceived. Prison officer staff perceived as “cold” Imported factors (modifiers brought in to prison by women) relating to self-harm behaviours such as past histories of abuse Situational factors (modifiers existing as a result of the women’s current situations) such as being on basic regime The purpose of self-harming behaviours was to control feelings of anger, frustration or a form of self-punishment
Marzano *et al.* (2011) England and Wales	Central Office for Research Ethics Committee (06/MRE12/83) Prison Service (PG 2006 063)	To identify socio-demographic, criminological and psychological variables associated with near-lethal self-harm. To provide further understanding of this behaviour and inform preventative initiatives. Mixed-methods (qualitative methods were descriptive, inferred)	Semi-structured, individual interviews Participants (*n* = 120) (60 with a history of self-harm, 60 with no history of self-harm) Audio recorded	Purposive sample of women who engaged in potentially lethal methods of self-harm	Interview location: Private room	Thematic analysis (no framework reported) N-Vivo (v8)	Negative relationships with staff were reported as antecedents to near-lethal self-harm. Being treated better by prison staff reported as a way to prevent self-harm behaviours in addition to not being in prison, having more distractions and time out of cell, more help with mental health problems, reduced access to means to self-harm, being in a shared cell and receiving counselling
Caulfield (2016) England	Associated University	To explore the suggestion that the prison environment exacerbates the incidence and severity of mental health issues. Qualitative descriptive (inferred)	Semi-structured, individual interviews Participants (*n* = 43) Audio recorded	Convenience sample using letters, posters, peer recruiters, prison officer recruiters, a prison television interview	Interview location: Not stated Privacy: only researcher and research participant present	Thematic analysis (no framework reported) N-Vivo	Positive experiences about prison-based health care included having access to therapy/prescriptions on a regular basis Being employed helped to reduce symptoms of depression Doctors advising that medication would suffice when counselling requested
Ahmed *et al.* (2016) Canada	University of Alberta Health Ethics Research Board	To report on female inmates’ perceptions of the barriers they face when accessing health care during incarceration, the impact this has on health during incarceration and in the community and implications for correctional health care practice. To show how these were used to inform the development of a resource aimed at improving functional health literacy regarding accessing correctional health care services Qualitative descriptive (inferred)	Focus groups Participants (n-12) Audio recorded	Purposeful sample from general correctional facility population meeting eligibility criteria for participation	Focus group location: Not stated	Thematic analysis (no framework reported). N-Vivo 10	Participants reported being unaware of services available to them because they did not see care being provided or because of poor health literacy Not being able to take a taxi to a medi-centre and manage self Being afraid of asking for help or further information when required Feeling “sloughed off” by staff Health care applications being sent back Having information provided that’s too simplistic and not reflective of the seriousness of the subject matter Reading other prisoners health care application forms
Jacobs and Giordano (2018) USA	All procedures in accordance with ethical standards of the national and/or institutional research committee and 1964 Helsinki Declaration	To illuminate service recipient’s perspectives on jail mental health care. To identify the elements of care most salient to participants while obtaining a unique perspective on service potential and limits. Qualitative inductive utilising techniques from Interpretative Phenomenology and Constructivist Grounded Theory	Semi-structured, individual interviews Participants (*n* = 19) Audio recorded	Purposeful sample of those who have a psychiatric diagnosis, had at least two experiences of jail detainment in the county, one of which occurring within two years of the interview	Interview location: Not stated	Techniques used in Constructivist Grounded Theory Techniques used in Interpretive Phenomenology	When medication regimes were adjusted this was mentally and physically harmful as well as discriminatory Assessment of mental health was overlooked on committal to prison No availability of “out of hours” crisis intervention services for prisoners

**Table 4:** Illustrative quotations organised around themes

Analytical themes	Quotations from participants in primary studies
1. The type of services accessed and challenges encountered	“We don’t even know about half the stuff that they offer here” (Ahmed *et al.*, 2016; p. 208). “I’m sure there could be a lot of great resources for woman but when you’re asking for it and don’t see it, it’s kind of … unbelievable. Yeah, it’s very discouraging and tiresome” (Ahmed *et al.*, 2016; p. 209). “I only started getting them 3 days after I came in. I had to wait for my medical records from GGGG [name of prison from where the prisoner had just been transferred] and until that came they couldn’t give us any medication. Thing is, I’d been on Methadone there … Yeah, it’s different in different goals … like in GGGG, if you’re on a scripts on the ‘out’, they give you what they call a ‘maintenance script’ inside, of a smaller dose. Whereas I was on 50 ml on the outside, so in GGGG I was getting 30 ml of Methadone and a sleeping tablet. And that was it. That was doing it. But when I came here, they told me they don’t do Methadone … they don’t give you sleeping pills. It’s a total no-no. So I was ill, very ill.” (Bowen *et al.*, 2009; p. 7). “So, it wasn’t until I actually got to prison and I was forced to take my pills that things started to look good. And I thought, oh no, if I’d have done this years ago, I wouldn’t be here now” (Caulfield, 2016; p. 222). “Helpful with [her] bi-polar. They gave me medications” (Jacobs and Giordano, 2018; p. 270). “When you need to see a doctor you have to put in an application, you have to wait too long and mainly because I’m quiet and because I don’t fuss” (Plugge *et al.*, 2008; p. e3). “So we put in a request form but it never gets taken care of, so then people get sick [or] mad and sad or their mental health issues get worse” (Ahmed *et al.*, 2016; p. 201)
2. A reduction in ability to self-manage mental well-being	“… we can’t you know, take a cab and go to a medi-centre down the road and go, you know what I mean, by ourselves.” (Ahmed *et al.*, 2016; p. 201). “the doctor said that anti-depressants would help me” (Caulfield, 2016, p. 223). “Because I’m on Valium-based medication – what everybody wants – I’m on Methadone. I kept giving a girl my Methadone all the time. She was bullying me into it” (Marzano *et al.*, 2011; p. 877). “I was on Trazadone and they [i.e. specifically the prison doctor] changed it to Venlafaxine … and that one I’ve forgot the name of, for the bi-polar, they just stopped them” (Bowen *et al.*, 2009; p. 4). “I didn’t bother. I was more concerned about taking my medication, and I didn’t want to say anything ‘cos I thought if I said anything they might just take me off it … So I just kept my mouth shut basically. I daren’t say anything. You know what they’re like” (Bowen *et al.*, 2009; p. 6). “He’s telling me he’s not giving no anti-depressants and I said “but I get it from my doctor” and he said “well, we’ll have to get in contact”… I’m telling him that antidepressants [are] what I take and he just says “no, no, no, you just want it because other people take it” (Plugge *et al.*, 2008; p. e4). “I’ve seen a psychiatrist for two-and-a-half, nearly three years I think, and he’s put me on medication, which I was quite happy to be on. It stopped when I come in here. Everything stopped and I’m a bit cheesed off” (Plugge *et al.*, 2008; p. e4). “I’ll just sit there thinking about my past, you know, how I’ve come to prison in the first place, I’ve let my kids down, you know and then I get really, really, really worked up and then I just have to see blood to, you know, relieve my pain” (Kenning *et al.*, 2010; p. 278)
2. The erosion of privacy and dignity	“I’ve read lots of request forms (giggle). I’m bad for it, but I mean like, if some girl has a sensitive issue why would she want to write that and let the guards read it [too]” (Ahmed *et al.*, 2016; p. 209). “I find it really upsetting because it’s just another indication of, that you’re not treated … as if you’re entitled to the same standard of health care that you would enjoy outside” (Plugge *et al.*, 2008; p. e4). “I’m serious about staying off drugs. I’m serious about all this stuff and I don’t want to sit here and read about ‘Sally and Joe’ and their little problems” (Ahmed *et al.*, 2016; p. 212)
4. Strained relationships between women and prison staff	“I just didn’t want to be around. I had enough of these [staff] pushing me and everything. I did” (Marzano *et al.*, 2011; p. 877). “They’re cold in their voice, they’re cold in the way they speak to you, they’re cold as in when you’re crying they just shut the door in your face” (Kenning *et al.*, 2010; p. 280). “Stop being stereotypical and thinking that we want drugs” (Plugge *et al.*, 2008; p. e5). “There’s so much coming and going, like people coming in, people leaving, people going to different wings, going to court, coming back – your file’s gone and it’s come back, it’s come up late. But I think some of it is not their fault, do you know what I mean, I’m not blaming it 100% on them” (Plugge *et al.*, 2008; p. e6)
